# Variations in Hyoid Kinematics Across Liquid Consistencies in Healthy Swallowing

**DOI:** 10.1044/2020_JSLHR-20-00508

**Published:** 2020-12-03

**Authors:** Sana Smaoui, Melanie Peladeau-Pigeon, Catriona M. Steele

**Affiliations:** a Swallowing Rehabilitation Research Laboratory, The Kite Research Institute - Toronto Rehabilitation Institute – University Health Network, Ontario, Canada; b Faculty of Medicine, Rehabilitation Sciences Institute, University of Toronto, Ontario, Canada

## Abstract

**Purpose:**

Judgments regarding hyoid movement are frequently included in evaluations of swallowing. However, the literature lacks reference values for measures of hyoid kinematics in healthy swallowing. This study explores hyoid movement across the continuum from thin to extremely thick liquids.

**Method:**

Participants were 39 healthy adults under the age of 60 years (19 men) who underwent videofluoroscopy involving three sips each of 20% w/v thin barium and six sips each of slightly, mildly, moderately, and extremely thick barium. Half of the thickened stimuli were prepared using xanthan gum; and half, with a starch-based thickener. Sip volume was derived from pre- and post-sip cup weights. Hyoid position was tracked frame-by-frame relative to the anterior–inferior corner of C4. Measures of peak hyoid position (along the *XY* axis) were normalized to a C2–C4 scalar, and measures of time-to-peak position, speed, and time-to-peak speed were derived. As a first step, Spearman's correlations confirmed the influence of sip volume on these hyoid measures. Linear mixed-effects models then explored the effects of stimulus, sip volume, and task repetition on the dependent variables.

**Results:**

The data set comprised 975 swallows with available hyoid tracking data. Sip volume was correlated with peak hyoid *XY* position (*r_s_* = .15, *p* < .01), time-to-peak position (*r_s_* = −.15, *p* < .05), and speed (*r_s_* = .13, *p* < .01). No significant differences in hyoid kinematics were found across stimuli.

**Conclusion:**

Measures of hyoid movement in healthy swallowing remain stable across the range from thin to extremely thick liquids with no systematic alterations in hyoid position or kinematics.

The hyoid is a floating bone that moves in response to suprahyoid muscle contraction with support and stabilization from the infrahyoid muscles including the thyrohyoid. Movement of the hyoid in an anterosuperior trajectory (i.e., the so-called “hyoid burst”) is frequently used to demarcate the onset of the pharyngeal phase of swallowing (Lof & Robbins, 1990; Pearson et al., 2011; Perlman et al., 1995). Hyoid movement is thought to play a role in facilitating safe and efficient swallowing and is, therefore, commonly investigated in clinical and instrumental swallowing assessments. Specifically, hyoid bone movement provides biomechanical advantages for laryngeal closure and airway protection (Steele et al., 2011) and is thought to generate traction forces that facilitate upper esophageal sphincter opening, enabling bolus clearance (Cook et al., 1989; Jacob et al., 1989; Mendelsohn & McConnel, 1987). The Mendelsohn maneuver, the Shaker head-lift exercise, the chin tuck against resistance exercise, and the recline exercise are all interventions that seek to optimize these biomechanical effects through greater excursion or prolonged duration of hyolaryngeal movement (Fujiki et al., 2019; Mendelsohn & McConnel, 1987; Shaker et al., 2002; Yoon et al., 2014).

Hyoid movement is commonly appraised using manual palpation during clinical swallowing examinations (McCullough et al., 2005) and using either visuoperceptual judgments (Martin-Harris et al., 2008; Perlman et al., 1994) or quantitative measures (Hind et al., 2001; Kim & McCullough, 2008; Molfenter & Steele, 2014) of videofluoroscopic swallowing study (VFSS) recordings. Recently, however, concerns have been raised regarding the validity of both palpation and visuoperceptually based judgments of hyoid excursion (Brates et al., 2019; Brates, Molfenter, & Thibeault, 2020). Palpation-based impressions of reduced peak superior hyoid position have been shown to agree with quantitative videofluoroscopic measurement, while impressions of reduced peak anterior or hypotenuse position were not confirmed. Visuoperceptual judgments of the peak hyoid position as normal or reduced on videofluoroscopy were also found to have poor agreement with quantitative measurement in all planes of movement. Quantitative measures of hyoid movement remain the gold standard as they reduce perceptual bias; however, the time required to make measurements is a barrier limiting uptake in clinical practice. Furthermore, interpretation of the adequacy of hyoid movement is currently hampered by a lack of healthy reference values for comparison.

Molfenter and Steele (2011) conducted a meta-analysis of studies reporting measures of hyoid movement on thin liquid swallows in healthy adults. Their results revealed a huge range in reported values of hyoid movement distance across the literature, with 95% confidence intervals ranging from 2 to 28 mm for superior displacement and from 6 to 19 mm for anterior displacement. In their discussion, Molfenter and Steele (2011) identified several methodological differences across studies that may have contributed to the high degree of observed variability (Dodds et al., 1988; Leonard et al., 2000; Logemann et al., 2000, 2002; Molfenter & Steele, 2011; Perlman et al., 1995). These included

1. differences in definitions, particularly with respect to the choice of “rest” frames used when measuring displacement between “rest” and peak positions, and also of the axes and coordinate systems used for measurement;
2. stimulus factors (bolus consistency and volume);
3. participant age and sex; and
4. the choice of units of measurement.

Regarding this last point, measures of peak hyoid position or displacement in millimeters have previously been reported to differ between men and women, with larger values seen in men (Logemann et al., 2000, 2002). This is not particularly surprising, given that men are, on average, taller than women and that the hyoid and larynx descend with puberty in males. However, Molfenter and Steele (2014) showed that normalizing hyoid measures to a cervical spine scalar, as previously suggested by Perlman et al. (1995), effectively neutralizes these sex differences. This finding was interpreted to show that hyoid movement is scaled to the size of the system, such that taller people with longer necks and longer pharynxes produce larger movements than shorter people. Molfenter and Steele (2014) recommended that measures of hyoid position or displacement be reported in anatomically scaled units (percentage of the length of the C2–C4 cervical spine), relative to an origin located at the anterior–inferior corner of the C4 vertebra, and using a coordinate system with the *y*-axis parallel to the C2–C4 spine and the *x*-axis derived orthogonal to the *y*-axis. Use of anatomical scaled measures also increases accuracy, by reducing measurement variability attributable to magnification and radial distortion (Sia et al., 2012).

Although measures of peak hyoid position or displacement are frequently reported, measures of hyoid movement timing can also be found in the literature (Lof & Robbins, 1990; Nagy et al., 2014, 2015). Lof and Robbins (1990) explored hyoid movement duration in healthy individuals, specifically measuring the duration of the interval when the hyoid is maximally displaced (durations of hyoid maximum anterior excursion and maximum superior elevation). Molfenter and Steele (2011) noted that hyoid movement duration was among the three most frequently reported timing intervals for measures of swallowing and drew attention to variability in the literature with regard to the operational definitions of temporal measurement. In particular, studies are not always clear whether measures of hyoid movement duration refer to specific portions of the hyoid movement (e.g., from burst to peak, or duration of time at peak position) versus the entire movement cycle from burst until return-to-rest. Hyoid movement timing may also be impacted by variations in bolus consistency, bolus volume, and cueing (Furkim et al., 2019).

Finally, although measures of hyoid displacement and timing are important, it has been proposed that derived measures of velocity, that is, change in position along a single axis, either superior (*Y*) or anterior (*X*) over time, or speed, that is, change in position along the XY hypotenuse over time, may tell a more informative story (Nagy et al., 2014, 2015; Zoratto et al., 2010). Peak hyoid velocity in the vertical plane has been reported to be significantly impacted by bolus volume (Nagy et al., 2014) and by bolus consistency (Nagy et al., 2015), with faster movement seen with larger boluses and thicker stimuli.

Steele et al. (2019) have recently published reference data for anatomically normalized measures of peak hyoid position (relative to the anterior–inferior corner of C4) in healthy adults under the age of 60 years on thin liquids and gum-thickened liquids. They did not find any significant effects of consistency for peak hyoid position along the *XY* axis. The goals of the current investigation were to extend this information by

1. establishing reference values for anatomically normalized measures of time-to-peak hyoid *XY* position and speed on thin liquid boluses and
2. confirming the presence/absence of differences in hyoid kinematics across stimuli, while controlling for variations in sip volume, with liquids ranging from thin to extremely thick consistency, according to the definitions of the International Dysphagia Diet Standardisation Initiative (IDDSI; Cichero et al., 2017), and thickened using both gum- and starch-based thickeners.

We adopted the null hypothesis, namely, that hyoid kinematics would remain stable, without evidence of significant variation across stimuli and without significant Sip Volume × Stimulus interactions.

## Method

This study was approved by the University Health Network Research Ethics Board (CAPCR #15-9431). The data for this article were collected as part of a larger study to establish reference values for videofluoroscopic measures of bolus flow and swallowing physiology across a range of consistencies (thin to extremely thick liquids). As described previously (Steele et al., 2019), videofluoroscopic data were collected from 39 healthy young adults (19 men) under 60 years old (*M*_age_ = 34 years, range: 21–58 years) with no history of swallowing, motor speech, gastroesophageal, or neurological difficulties or major surgery or radiation to the head or neck. Videofluoroscopies were recorded in lateral view using the KayPENTAX Digital Swallowing Workstation at an image acquisition rate of 30 images per second. Low-concentration barium stimuli (20% weight/volume) were prepared using Bracco Diagnostics E-Z-PAQUE powdered barium sulfate, bottled water (Nestlé Pure Life), and either a xanthan gum–based thickener (Resource ThickenUp Clear, Nestlé Health Science) or a starch-based thickener (Resource ThickenUp, Nestlé Health Science). Regardless of thickener type, stimuli were prepared according to standard recipes (Barbon & Steele, 2019), to meet the flow characteristics of each liquid level of the IDDSI framework (Cichero et al., 2017): thin, slightly thick, mildly thick, moderately thick, and extremely thick. Three cups, each containing 40 ml of a given stimulus, were prepared for a total of 27 stimuli (3 × thin and three each of consistency-plus-thickener combination). The stimuli were served in blocks of increasing thickness, with the gum-thickened block of each consistency preceding the starch-thickened block. The thin, slightly thick, and mildly thick liquids were taken by comfortable sip without a command to swallow. The moderately and extremely thick liquids were taken by teaspoon, again without a command to swallow. Measures of sip volume were derived using pre- and post-sip cup weights. The VFSS recordings were stripped of audio, spliced into individual bolus level clips, de-identified, and randomized for VFSS rating.

### Videofluoroscopy Analysis

**Figure 1.**
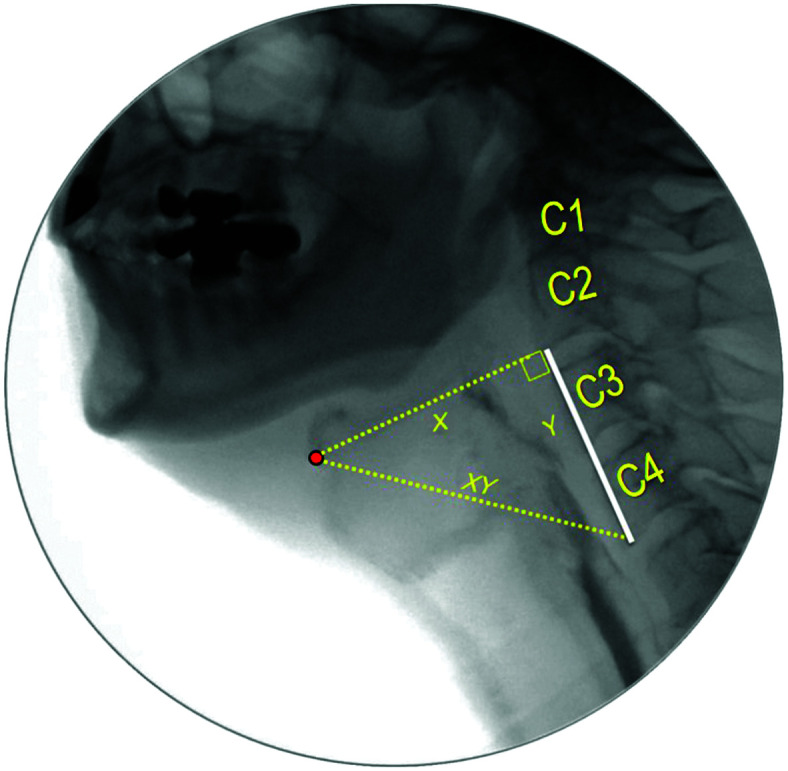
Videofluoroscopic swallowing study image illustrating the coordinate system used for hyoid bone tracking. The red dot represents the marker placed at the anterior–inferior corner of the hyoid bone to track its kinematics.

Hyoid position tracking was performed by trained raters using ImageJ open source software (National Institutes of Health). For the current analysis, the interval of interest began 5 frames prior to onset of the hyoid burst movement for the initial swallow of each bolus and continued until 5 frames after hyoid movement had begun in its descent from peak position for that same swallow. For each frame, the following structures were marked: (a) the anterior–inferior corner of the C4 vertebra (origin of the coordinate system), (b) the anterior–inferior corner of the C2 vertebra (*y* plane), and (c) the anterior–inferior corner of the hyoid bone (see Figure 1). Using these marked points, the *X*, *Y* and *XY* coordinates of hyoid position, relative to the C4 origin, were calculated in anatomically normalized units, that is, %(C2–4) (Molfenter & Steele, 2014). In cases where the hyoid moved outside the field of view, or where the shoulder or other structures obstructed the view of the cervical spine, a missing datapoint was recorded.

The *XY* hyoid position histories were inspected to identify the frame of peak *XY* position identified as the frame of greatest excursion along that plane. Time-to-peak *XY* position was calculated by counting the number of frames between the frame of peak *XY* position and the frame of hyoid burst onset. Hyoid *XY* speed was calculated by dividing the difference in hyoid *XY* position between the frames of peak *XY* position and hyoid burst onset by time-to-peak *XY* position.

### Statistical Analysis

All statistical analyses were conducted using IBM SPSS Statistics Version 26. Descriptive statistics were calculated for all hyoid parameters and sip volume. Continuous variables were inspected using q–q plots and histograms to examine normality; this revealed nonnormal distributions of residuals for time-to-peak position and speed. A rank-based transformation based on proportion estimates was applied to these parameters to control for Type 1 error due to asymmetrical residual distribution using Blom's formula (Blom, 1954). As a first step, Spearman's rank correlations were used to examine the relationships between sip volume and the hyoid parameters and to determine whether sip volume should be considered as a covariate in the planned statistical models. Linear mixed-model analyses with a compound symmetry structure were then performed with a factor of stimulus (thin; slightly thick gum, slightly thick starch; mildly thick gum, mildly thick starch; moderately thick gum, moderately thick starch; extremely thick gum, extremely thick starch), a covariate of sip volume, a random effect of participant, and a repeated measure of task repetition. Where significant effects were found, post hoc Sidak tests were used to explore pairwise comparisons. Statistical significance for the linear mixed models was set for *p* values < .05.

## Results

The data set was composed of 975 swallows with available hyoid tracking data. Descriptive statistics appear in Tables 1 [Table T2]–3. Spearman's rank correlation analyses revealed weak but statistically significant correlations between sip volume and (a) peak hyoid *XY* position (*r_s_* = .15, *p* < .05), (b) time-to-peak position (*r_s_* = −.15, *p* < .05), and (c) hyoid speed (*r_s_* = .13, *p* < .05). Given these findings, sip volume was carried forward into the linear mixed models as a covariate.

**Table 1.** Hyoid peak *XY* position by stimuli.

Hyoid peak *XY* position (%(C2–4))						
Stimulus			95% confidence interval		Quartiles	
	*M*	*SD*	Lower bound	Upper bound	25%	75%
IDDSI 0 (thin)	169	16	165	172	162	176
IDDSI 1 (slightly thick)						
Starch	169	16	165	171	161	179
Gum	168	18	165	172	160	181
IDDSI 2 (mildly thick)						
Starch	168	15	166	171	160	178
Gum	170	16	167	173	163	181
IDDSI 3 (moderately thick)						
Starch	168	15	165	170	159	178
Gum	168	18	165	171	157	180
IDDSI 4 (extremely thick)						
Starch	170	14	167	173	163	179
Gum	168	15	166	172	162	181

*Note.* IDDSI = International Dysphagia Diet Standardisation Initiative.

**Table 2. T2:** Hyoid time-to-peak position by stimuli.

Hyoid time-to-peak-position (ms)						
Stimulus			95% confidence interval		Quartiles	
	*M*	*SD*	Lower bound	Upper bound	25%	75%
IDDSI 0 (thin)	393	123	369	418	300	467
IDDSI 1 (slightly thick)						
Starch	384	121	361	407	300	467
Gum	376	114	354	398	300	467
IDDSI 2 (mildly thick)						
Starch	391	137	365	417	267	467
Gum	385	130	361	409	300	467
IDDSI 3 (moderately thick)						
Starch	403	108	383	423	334	467
Gum	395	111	374	417	300	467
IDDSI 4 (extremely thick)						
Starch	408	91	390	425	334	467
Gum	410	120	386	433	333	500

*Note.* IDDSI = International Dysphagia Diet Standardisation Initiative.

**Table 3.** Hyoid *XY* speed by stimuli.

*XY* speed (%(C2–4)/s)						
Stimulus			95% confidence interval		Quartiles	
	*M*	*SD*	Lower bound	Upper bound	25%	75%
IDDSI 0 (thin)	124	43	116	133	96	150
IDDSI 1 (slightly thick)						
Starch	123	42	115	131	95	145
Gum	125	45	116	134	96	150
IDDSI 2 (mildly thick)						
Starch	123	49	114	132	91	151
Gum	128	54	117	138	93	146
IDDSI 3 (moderately thick)						
Starch	112	37	105	119	88	131
Gum	114	36	107	121	90	139
IDDSI 4 (extremely thick)						
Starch	116	34	109	122	91	134
Gum	121	46	112	130	88	147

*Note.* IDDSI = International Dysphagia Diet Standardisation Initiative.

### Linear Mixed-Model Analyses

**Figure 2.**
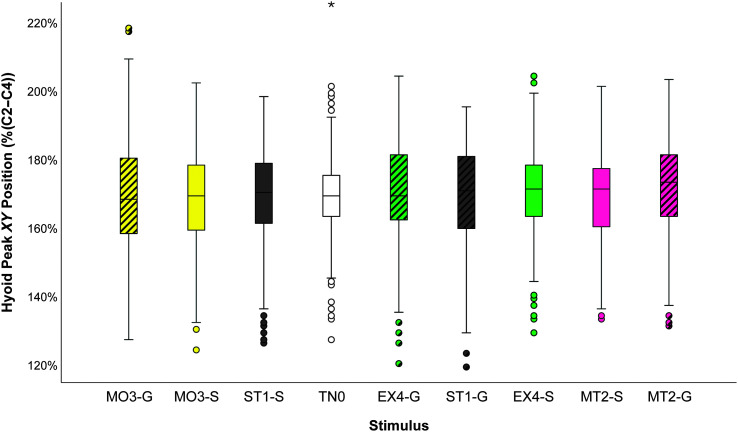
Box plots of peak *XY* hyoid position by stimulus, with stimulus consistency coded and colored according to the International Dysphagia Diet Standardisation Initiative framework. EX4 = extremely thick; -G = gum-thickened; MO3 = moderately thick; MT2 = mildly thick; -S = starch-thickened; ST1 = slightly thick; TN0 = thin.

Linear mixed models exploring the influence of a number of variables (stimulus, administration method, and sip volume) on hyoid peak *XY* position (%(C2–4)) revealed a significant interaction for the factor of stimulus by the covariate of sip volume, *F*(8, 887.67) = 2.61, *p* = .008. Additionally, main effects were found each for stimulus, *F*(8, 886.35) = 2.44, *p* = .013, and sip volume, *F*(1, 921.26) = 8.78, *p* = .003. No other relationships reached statistical significance for hyoid peak *XY* position. A box plot illustrating peak *XY* position, ranked in ascending order of mean values, can be found in Figure 2. Pairwise comparisons using the Sidak test failed to identify any significant differences between paired stimuli. With regard to time-to-peak position (milliseconds), no significant main effects, covariate effects, or interactions were identified in the linear mixed model. Linear mixed models exploring effects on rank-normalized hyoid *XY* speed (%(C2–4) per second) revealed a significant main effect for sip volume, *F*(1, 673.82) = 9.63, *p* = .002, consistent with the previously reported Spearman correlations.

## Discussion

This study explores differences in hyoid kinematics in healthy adults with gum- and starch-thickened stimuli representing the continuum of IDDSI liquid levels. Our explorations using linear mixed-model analyses reveal that peak hyoid *XY* position, time-to-peak *XY* position, and *XY* speed do not vary significantly across liquid stimuli with different consistencies in healthy adults.

Hyoid data have historically been extracted using two different methods: either in absolute units (millimeters) relative to an external coin scalar or referenced to an internal anatomical scalar. The method of using the internal anatomical scalar reduces variability by normalizing the movement to the individual participant and controls for sex-based differences. Our rating method utilized the C2–C4 length as a scalar, which is consistent with the methods used in recent literature (Brates, Steele, & Molfenter, 2020; Molfenter & Steele, 2014; Nagy et al., 2014, 2015). The values presented in Tables 1, 2, and 3 can therefore be compared to data reported in these studies.

The hyoid peak *XY* position data in Table 1 show a mean value of 169%(C2–4) for the thin liquid consistency. This value is larger than the values reported by Molfenter and Steele (2014) for each sex, where they report values ranging from 148.2 to 157.9 %(C2–4) in men and from 147.7 to 153.0 %(C2–4) in women. Our values are also larger than those reported by Brates, Steele, and Molfenter (2020) for ultrathin and thin liquid barium consistencies, ranging from 147.6 to 153.3 %(C2–4). It is important to note, however, that both of these prior studies used controlled bolus amounts, while participants in our studies took uncontrolled comfortable cup sips, with the resulting data demonstrating larger peak hyoid position values for larger sip volumes. The values reported in the Molfenter and Steele (2014) and Brates, Steele, and Molfenter (2020) studies for the 20-ml thin consistency condition are similar to the values seen in our data for the thin consistency condition. The time-to-peak position values in our study are slightly shorter for both the thin and mildly thick consistencies compared to those reported by Nagy et al. (2015). Again, differences in bolus volume control across studies may be responsible.

Calculations of hyoid *XY* speed are referred to by Nagy et al. (2014, 2015) as “hyoid burst movement velocity along the *XY* axis.” In their 2014 article, Nagy et al. report *XY* hyoid velocity to have a mean value of 49 mm/s (95% CI [45, 53]) on thin liquids ranging from 5 to 20 ml in volume. Unfortunately, these values, reported in units of millimeters per second, cannot be compared to the %(C2–4)/s units used in our study. In their subsequent article, Nagy et al. (2015) report *XY* velocity data in %(C2–4) units per second for ultrathin, thin, and nectar-thick liquids, with mean values of 116 %(C2–4)/s, 110 %(C2–4)/s, and 137 %(C2–4)/s, respectively, for nectar-thick liquids. These values are comparable to the mean values in our data set of 124 %(C2–4)/s (thin), 123 %(C2–4)/s (starch), and 128 %(C2–4)/s (gum) for the IDDSI 2 (mildly thick liquid) liquids.

As noted in the Method section, participants in this study were instructed to sip the thin, slightly thick, and mildly thick stimuli but to use a spoon to serve the moderately and extremely thick stimuli for all trials. The spoon administration instructions limited the volume of material that the participants took for the thicker consistencies and may have impacted our dependent variables. Although we found correlations between sip volume and consistency, these correlations were weak and appeared to impact the moderately and extremely thick stimuli more than the other stimuli, increasing the likelihood that this may have been an artifact of the spoon administration method. As demonstrated by the box plot in Figure 2, the highest mean values for hyoid peak *XY* position were seen for mildly thick liquids, which were sipped and had the largest volumes. Moderately thick liquids, however, had the lowest mean values and the smallest sip volumes. This sip volume by stimulus trend was later confirmed in the linear mixed models.

This study included flow-matched stimuli in accordance with the IDDSI framework using both a gum-based thickener and a starch-based thickener for each consistency level. Although the viscosities of the starch-thickened stimuli were considerably higher than those of the gum-thickened stimuli (Ong et al., 2018), our analyses did not find any differences in hyoid measures between stimuli as a function of thickener type. The range of movement and the timing of these movements in healthy adults can, therefore, be understood to remain stable, regardless of the different stimuli that were presented. The implication of our findings for clinical practice is that variations in hyoid timing, position, and speed should not be attributed to stimulus consistency or thickener type, particularly if they fall outside the reported reference values.

The analysis conducted in this study focused on healthy young adults aged below 60 years, which limits the generalizability of these results beyond this age range. Clinicians utilizing these normative values should proceed with caution when performing comparisons with patient populations outside of this age range. The methodology used in this analysis may also pose barriers to uptake in clinical practice due to the training and time commitment needed to perform frame-by-frame hyoid movement tracking. Research continues to develop in the area of automated hyoid bone movement detection (Lee et al., 2020; Zhang et al., 2018), which may allow clinicians to derive these hyoid measures using machine learning software in the future.

In contrast to the methods utilized in this article, hyoid trajectories have historically been described in the literature across a single plane of movement—superior or anterior. The current methods consider hyoid trajectories along the hypotenuse plane that incorporates the anterior and superior components of the movement, allowing for a comparison of peak position and speed across individuals. It would be interesting for future studies to investigate whether there are different hyoid trajectories, for example, single vector movements that track along the hypotenuse versus those with a biphasic pattern (e.g., initial superior movement followed by an anterior movement). Confirming whether the stability of hyoid measures seen in this article remains true when hyoid movement pattern is considered would be valuable information.

## Conclusions

In this study of 39 healthy adult participants, we established reference values for measures of peak hyoid *XY* position, time-to-peak hyoid *XY* position, and hyoid *XY* speed. Our analyses confirmed relatively stable hyoid timing and kinematics across different liquid consistencies and thickener types. Prior studies have identified disordered hyoid movement in individuals with dysphagia; however, the impact of varying stimuli and their effect on this movement have not been considered in a healthy cohort. These data allow for a clinical comparison with healthy reference data, indicating a range in which hyoid movement can be considered within normal limits. Future research exploring swallowing physiology and hyoid parameters across controlled bolus volumes of different textures may further clarify the role of volume on changes in hyoid parameters.

## Author Contributions

- Sana Smaoui: Conceptualization (Equal), Data curation (Equal), Formal analysis (Equal), Investigation (Equal), Methodology (Equal), Resources (Equal), Validation (Equal), Visualization (Lead), Writing – original draft (Lead), Writing – review & editing (Equal).
- Melanie Peladeau-Pigeon: Data curation (Equal), Investigation (Equal), Methodology (Equal), Project administration (Equal), Resources (Equal), Software (Lead), Writing – review & editing (Supporting).
- Catriona M. Steele: Conceptualization (Equal), Formal analysis (Equal), Funding acquisition (Lead), Investigation (Lead), Methodology (Equal), Project administration (Equal), Supervision (Lead), Validation (Lead), Writing – original draft (Equal), Writing – review & editing (Equal).
